# The Anterior Interosseus Artery Perforator Flap: Anatomical Dissections and Clinical Study

**Published:** 2017-05

**Authors:** Nikhil S Panse, Sheetal B Joshi, Parag B Sahasrabudhe, B Bahetee, Pradnya Gurude, Ajay Chandanwale

**Affiliations:** 1 Department of Plastic Surgery, BJGMC, Pune, India;; 2 Department of Anatomy, Lady Hardinge Hospital, New Delhi, India;; 3 Department of Anatomy, BJGMC, Pune, India;; 4 Department of Orthopedics, BJGMC, Pune, India

**Keywords:** Upper extremity, Perforator flap, Propeller flap, Anterior interosseus artery perforator flap

## Abstract

**BACKGROUND:**

Reconstruction of upper extremity deformities continues to be a challenge to the reconstructive surgeon. Various loco regional, distant and free flaps are available for reconstruction. However, each has its own set of advantages and disadvantages. Of the commonly performed local flaps, radial artery forearm flap, and the posterior interosseus artery flap stand out prominently. Recently, perforator propeller flaps have been used for resurfacing the upper extremity. The anterior interosseus artery perforator flap is an uncommonly used and described flap.

**METHODS:**

This study was divided into anatomical study and clinical application in a IV level of evidence. In the anatomical study, five upper extremities were studied. Clinically, 12 patients underwent reconstruction using the anterior interosseus artery perforator flap. Flaps were performed by a single surgeon. A retrospective review of these cases from November 2008 to May 2014 is presented.

**RESULTS:**

The anterior interosseus artery perforator was identified in four out of five cadaver limbs. The septocutaneous perforator was in the fifth extensor compartment around 4 cm proximal to the wrist joint. Of the twelve flaps, there was complete necrosis in one flap, and partial necrosis in one flap. The patient with complete necrosis underwent skin grafting at a later date. The wound healed secondarily in case of partial flap necrosis.

**CONCLUSION:**

Anterior interosseus artery perforator flap must be considered as an important reconstructive option in the armamentarium of the plastic surgeon, while managing hand and wrist defects.

## INTRODUCTION

Soft tissue defects of the hand can be caused by various reasons like trauma, tumor resection, and scar release. In case the vital structures are exposed, flap cover is needed. Various loco regional options have been described in literature for coverage of these defects, with their own set of advantages and disadvantages.^1^^-^^5^ Final decision making regarding flap cover is based on preference of the patient, and comfort of the surgeon to execute that particular flap. The literature on the use of the anterior interosseus artery perforator flap is extremely sparse. We present our anatomical study and clinical applications of the anterior interosseus artery perforator flap. 

## MATERIALS AND METHODS

**Fig. 1:**
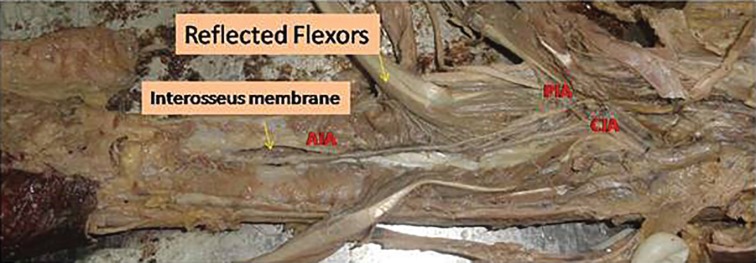
Identification of the anterior interosseus artery.

**Fig. 2:**
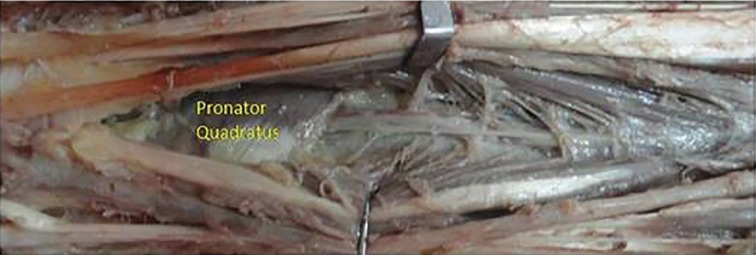
Anterior interosseus artery and Pronator Quadratus muscle

**Fig. 3:**
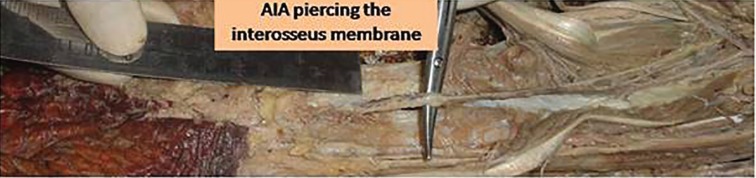
Anterior interosseus artery perforating the interosseus membrane.

Cadaver dissection with dye study was performed in five cadavers to study the anatomy of the anterior interosseus artery perforator. A volar incision was made, flexor tendons retracted, and transected wherever necessary to gain access to the anterior interosseus artery. The anterior interosseus artery was identified after its branching from the common interosseus artery (Figure 1). It was traced distally to the level, where it gives a branch and goes beneath the pronator quadrates (Figure 2 and 3). The anterior interosseus artery was transected, ligated proximally, and a neocath was inserted in its distal end. Dorsal skin flaps were raised in the subcutaneous plane to identify the septocutaneous perforators coming dorsally. 

To additionally confirm the perforators, 2-3 cc of methylene blue was gradually injected though the anterior interosseus artery and its perforators on the dorsum were identified. Between November 2008 and May 2014, twelve patients underwent reconstruction using the anterior interosseus artery perforator flap. Flaps were performed by the first author for various locations and indications over the upper extremity. Retrospective analysis of patient demographics, surgical indication, defect features, complications and clinical outcomes are presented. Patients with history of smoking, diabetes and other co morbidities were included in the group. Extremities with extensive trauma zone and degloving were excluded.

Akin performed two anterior interosseus artery perforator flaps in VY fashion for hand defects, and also reviewed the anatomy of the anterior interosseus artery.^6^ The anterior interosseus artery arises from the common interosseus artery that originates from the ulnar artery. It runs on the volar surface of the interosseus membrane.^7^^-^^10^ After sending a branch to the pronator quadratus muscle, the anterior interosseus artery gives off a perforator that pierces the interosseus membrane approximately 5 cm proximal to the distal radioulnar joint.^7^^,^^8^^,^^10^ The terminal branch of the anterior interosseus artery makes anastomoses with branches of the ulnar and radial arteries at the palmar aspect of the wrist.^7^^-^^10^

The perforator that is accompanied by two venae comitantes passes to the dorsal aspect of the forearm and gives off several small branches to the extensor muscles of the thumb and distal third of the radius.^7^^,^^10^ The perforator enters the dorsal forearm fascia and then bifurcates into proximal and distal branches.^7^ These two branches together supply the distal two-thirds of the dorsal forearm.^7^^-^^10^ The distal branch anastomoses with a branch of the posterior interosseus artery at the level of the wrist joint. Furthermore, it also makes several anastomoses with cutaneous branches of the ulnar and radial arteries at the dorsal aspect of the wrist. The perforator is approximately 4 to 6 cm in length and 0.9 to 1.6 mm in diameter.^7^^-^^10^

In patient selection, small to moderate sized defects can be effectively managed by anterior interosseus artery perforator flap. Patients with multiple fractures to the extremity, degloving injuries, and trauma zone in the area of perforator are not considered for the anterior interosseus artery perforator flap.^5^ We consider preoperative Doppler as desirable and not mandatory prerequisite for performing an anterior interosseus artery perforator flap. The Doppler study is made with a hand held Doppler with an 8 Hz frequency probe to identify the perforator. We prefer a partially exsanguinated extremity as it eases the identification of the perforators.

An ulnar sided incision over the dorsum of the wrist is made, extending upwards from about 1-1.5 cm proximal to the wrist joint. Skin flaps are raised to identify the perforator of the anterior interosseus artery. Once the perforator is identified, found to be of adequate caliber, and free of zone of trauma, flap is planned. The distance of the perforator to the distal edge of the defect is measured. Planning is made in reverse, considering the degree of rotation involved, and distal edge of the flap is marked along the long axis of the upper extremity on the line joining the lateral epicondyle and the distal radioulnar joint. 

Due care is taken to add 1-1.5 cm to the long axis of the flap. The width of the defect is noted and taken into consideration while planning. A tail like extension of the flap is made upto the pivot point. This prevents compression of the pedicle and facilitates tension and pressure free closure of the pedicle after exteriorisation. The flap is then harvested subfascially or suprafascially, and islanded on the perforator. The decision to harvest the flap sub fascially or suprafascially is made as mentioned by Panse *et al.*^5^

All fibrous strands are dissected to prevent compression on the perforator after rotation. It is generally not necessary to dissect the perforator till the interosseus membrane. A subcutaneous vein is always preserved at the base of the flap. Throughout the procedure, a lignocaine soaked small piece of gauze is kept over the perforator. The perforator/guaze is irrigated by lignocaine solution to prevent drying and spasm of the perforator.^5^^,^^11^ Once the flap is islanded, tourniquet is released and flap is permitted to perfuse for a while before rotation. Cautery is used judiciously as and when needed away from the perforator to achieve absolute hemostasis.

**Fig. 4:**
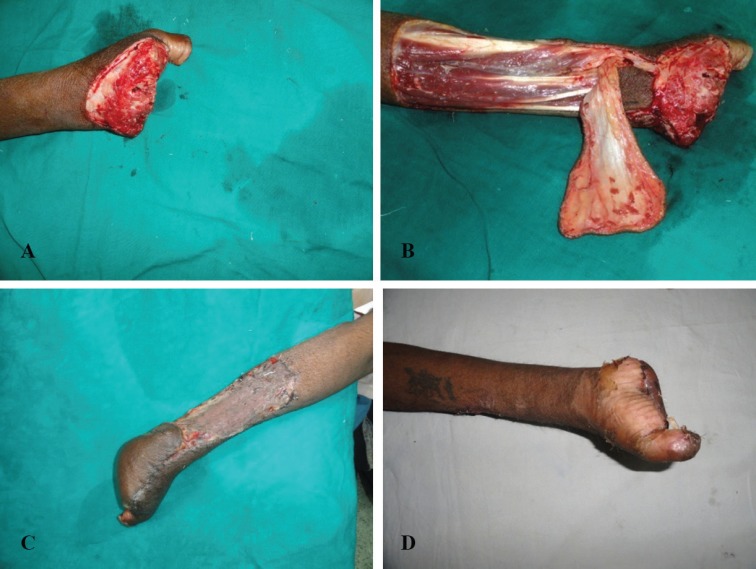
Anterior interosseus artery perforator flap for amputation stump coverage. A) Post traumatic amputation at the MCP level. B) Harvested anterior interosseus artery perforator flap on the cutaneous perforator of the anterior interosseus artery just proximal to the wrist joint. (Please note absence of posterior interosseus artery on the undersurface of the flap) C) Well settled flap. D) Palmar aspect of the hand showing distal aspect of the flap

**Fig. 5:**
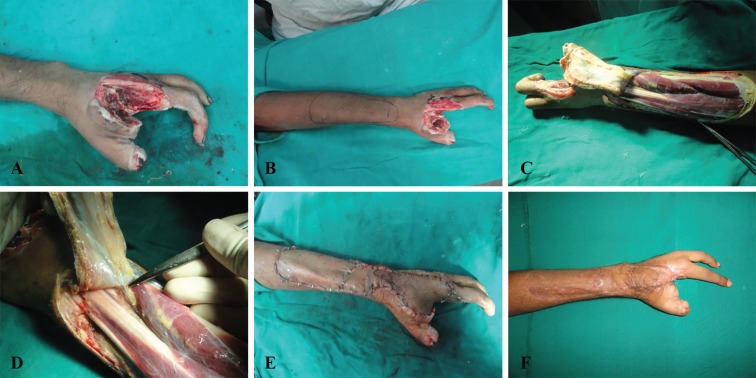
Anterior interosseus artery perforator flap for dorsum hand coverage. A) Defect with exposed metacarpals. B) Perforator and Flap markings. C) Harvested anterior interosseus artery perforator flap on the cutaneous perforator of the anterior interosseus artery just proximal to the wrist joint. (Please note absence of posterior interosseus artery on the undersurface of the flap) D) Perforator. E) Flap in situ. F) Partial necrosis of flap with secondary healing, and well settled donor site

The flap is then propelled as necessary to cover the defect. The initial sutures are taken along the sides of the perforator to prevent traction to the perforator.^5^^,^^11^ Due care is taken to inset the flap without any tension. The flaps appear a bit congested in the initial few days, but gradually settle down with time. Few clinical cases are shown (Figure 4 and 5). Splintage and strict limb elevation is maintained for a period of ten days. Arm slings are generally avoided for the initial couple of days. If they are applied, they are applied in a fashion that does not cause compression over the perforator. Crepe bandage is initiated on day 7. Sutures are removed on 10-12^th^ day, and physiotherapy as and where necessary is initiated.

## RESULTS

**Fig. 6:**
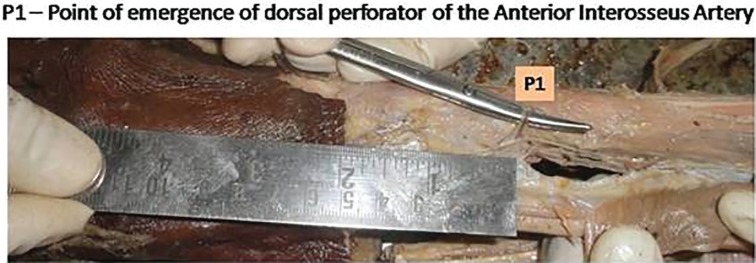
Anterior interosseus artery perforator at 4cms from the wrist

A single constant perforator was identified coming out of the fifth extensor compartment, between the ECU and EDM. The perforator was relatively constant at around four cms proximal to the wrist joint (Figure 6). In one cadaver, there was no perforator which was identified on the dorsum of the wrist. Details of cadaver dissection provided in Table 1. The anterior interosseus artery perforator flap was performed in 12 patients between November 2008 and May 2014. Of the 12 patients, 8 were male, and 4 were female. The age group ranged from 9 to 51 years. Nine Flaps were performed for trauma, and 2 were performed for post burn contracture release. 

**Table 1:** Perforator locations in cadaver dissections

**Sr. No**	**No. of perforators**	**Extensor compartment**	**Distance proximal to wrist joint (cm)**
1.	1	5^th^	3.8
2.	1	5^th^	4
3.	1	5^th^	4.2
4.	0	0	0
5	1	5^th^	4

Of the nine patient of trauma, flap was performed in four patients for amputation stump coverage, three patients for dorsum hand coverage, one for proximal palmer resurfacing, and one for distal wrist resurfacing. The defect sizes ranged from 3×4 cm to 6x10cms. The follow up ranged from three weeks to six months. All the donor sites necessitated skin grafting. Of the twelve flaps, there was complete necrosis in one flap, and partial necrosis in one flap. The patient with complete necrosis underwent skin grafting at a later date. The wound healed secondarily in case of partial flap necrosis. Both the flaps were lost to congestion.

## DISCUSSION

A wide variety of loco regional flaps are available to us for resurfacing of hand defects. Each of them has its own set of advantages and disadvantages. Pedicle distant flaps like abdominal and groin flaps are reliable and safe. But it requires prolonged immobilization resulting in stiffness of uninvolved joints. The bulk is excessive and repeated debulking surgeries are almost always the rule. Free flaps are a good option for medium to large defects with a variety of donor options to suit the requirements of the particular patient. However they are time consuming, infrastructure dependent, have a significant learning curve and have a potential for total flap loss. Moreover, facilities are not universally available. 

Distally based island fasciocutaneous flaps in the forearm have proved to be simple, versatile and reliable for soft tissue reconstruction of the hand.^4^ The more commonly used ones are the reverse radial, reverse ulnar and the posterior interosseus artery flaps. The radial and the ulnar forearm flaps are the most popular flaps for soft tissue cover of the hand as with their own set of disadvantages.^1^^,^^2^^,^^4^^,^^12^ Both flaps are based on the integrity of the palmar arches and a major artery for the vascular supply of the hand is sacrificed by flap harvest. Jones *et al.*^13^ and Timmons *et al.*^14^ reported cold intolerance after harvestation of the radial forearm flap. Needless to mention, it is obvious that the radial and ulnar arteries are more important than the posterior interosseus artery flap. 

The posterior interosseus artery flap has got its own set of disadvantages. There is enormous literature on the posterior interosseus artery flap. The anatomical variability of the anastomosis must be considered. Costa *et al.*^4^ encountered one clinical case where there was bilateral disappearance of the posterior interosseus artery in the middle third of the forearm. Their combined cadaveric and clinical series shows that in 2 cases, elevation of a distally based posterior interosseus island flap would not have been possible.^4^ In a series of 40 fresh cadaver specimens and 80 clinical cases of posterior interosseus reverse forearm flaps, Angrigiani *et al.*^15^ noted absence of the continuity of the posterior interosseus artery at the level of the mid-forearm in one anatomic specimen and one clinical case. They also observed narrowing of the posterior interosseus artery in the midforearm in 74 out of 80 clinical cases. 

Giunta and Lukas^16^ reported a clinical case of absence of continuity of the posterior interosseous artery in the middle third of the forearm. The Posterior interosseus Artery flap dissection is tedious, plagued with the chances of anatomical variations and there are chances of injuring the posterior interosseus nerve during flap harvest. Hubmera *et al.*^17^ have extensively studied the anatomy of the posterior interosseus artery, and anterior interosseus artery to its termination. In their dissection study on 66 cadavers, they noted that The Anterior Interosseus Artery emerges on the dorsal face of the interosseus membrane 44 mm (30–55 mm) proximal to the styloid process. 

Although the branch through the fifth extensor compartment, was found in all their specimens, and seemed to continue the course of the dorsal branch of the anterior interosseus artery, the authors believed that this vessel is a branch originating from the vascular arch.^17^ Sheetz *et al.*^18^ in their study of arterial blood supply of distal radius and ulna encountered branches of the anterior interosseus artery through the fourth extensor compartment. We studied only five cadavers, and the dorsal branch of the Anterior Interosseus Artery was present in four of those, which emerged through the fifth compartment. In one case, we did not encounter any branch coming out dorsally.

The anterior interosseous artery flap has been previously reported by Martin, Hu, Shibata, and Syed.^7^^-^^10^ They raised the anterior interosseus flap in a plane between muscle and fascia and divided the interosseus membrane to increase the pedicle length.^11^^,^^13^^,^^14^ Akin^6^ elevated an anterior interosseus flap as a V-Y advancement island flap from the dorsal aspect of the forearm for the dorsal defects of the wrist. This flap was based on the dorsal perforator of the anterior Interosseus artery. We too elevated this flap on the dorsal perforator of the anterior interosseus artery, and propelled it to cover defects up to the metacarpal heads on the dorsum, volar aspect of the wrist, as well as proximal half of the palm.

The anterior interosseus artery perforator flap has all the advantages of the posterior interosseus artery flap without any of its disadvantages. The area of flap harvest is the same. Since the Posterior Interosseus Artery is not included in the flap, there are no chances of injuring the posterior interosseus nerve. Moreover, it has got nothing to do with the numerous anatomical variations of the posterior interosseus artery. If a perforator can be identified on exploratory incision, the flap can be raised safely without having to dissect the perforator till the interosseus membrane. The operative time is reduced substantially, and there is no sacrifice of one of the major limb vessels. This flap can be raised, even if there is no continuity of the dorsal arch, especially in case of amputation stump coverage distal to the wrist joint, and through the palmar arch.

One of the major disadvantages of the Anterior Interosseus Artery Perforator flap is its unsightly donor site over the exposed forearm. However, it is true for all the forearm flaps. The anterior interosseus artery perforator flap is a versatile flap. More number of anatomical and clinical studies are needed to establish the safety and efficacy of this flap. However, our preliminary experience is encouraging, and we feel that this flap can be the workhorse forearm flap for hand resurfacing.
